# Production of Micro- and Nanoscale Lignin from Wheat Straw Using Different Precipitation Setups

**DOI:** 10.3390/molecules23030633

**Published:** 2018-03-11

**Authors:** Stefan Beisl, Petra Loidolt, Angela Miltner, Michael Harasek, Anton Friedl

**Affiliations:** Institute of Chemical, Environmental and Bioscience Engineering, TU Wien, 1060 Vienna, Austria; petra.loidolt@gmx.at (P.L.); angela.miltner@tuwien.ac.at (A.M.); michael.harasek@tuwien.ac.at (M.H.); anton.friedl@tuwien.ac.at (A.F.)

**Keywords:** lignin, lignocellulose, nanoparticles, microparticles, biorefinery, organosolv, wheat straw, direct precipitation

## Abstract

Micro- and nanosize lignin has recently gained interest due to its improved properties compared to standard lignin available today. As the second most abundant biopolymer after cellulose, lignin is readily available but used for rather low-value applications. Applications for lignin in micro- to nanoscale however, ranging from improvement of mechanical properties of polymer nanocomposites, have bactericidal and antioxidant properties and impregnations to hollow lignin drug carriers for hydrophobic and hydrophilic substances. This research represents a whole biorefinery process chain and compares different precipitation setups to produce submicron lignin particles from lignin containing an organosolv pretreatment extract from wheat straw. A batch precipitation in a stirred vessel was compared with continuous mixing of extract and antisolvent in a T-fitting and mixing in a T-fitting followed by a static mixer. The precipitation in the combination of T-fitting and static mixer with improved precipitation parameters yields the smallest particle size of around 100 nm. Furthermore, drying of particles did not influence the particle sizes negatively by showing decreased particle diameters after the separation process.

## 1. Introduction

Lignocellulosic biomass residues are estimated to exceed 2 × 10^11^ t/year worldwide and offers a vast source for lignin [1]. The major part of the lignin is used as an energy source. Considering a lignocellulose biorefinery producing bioethanol, only around 40% of the produced lignin is needed to cover the thermal energy demand [2,3]. Hence, 60% of the generated lignin is available to maximize valorization in addition to the valorization of the carbohydrate fractions. This increased valorization is necessary to improve the utilization of the entire biomass and therefore to enhance the economical value [4].

Lignin is a highly irregular branched polyphenolic polyether, consisting of the primary monolignols, p-coumaryl alcohol, coniferyl alcohol and sinapyl alcohol, which are connected via aromatic and aliphatic ether bonds [5]. Roughly three different types of lignins can be distinguished: softwood lignins are comprised almost solely of coniferyl alcohol, hardwood lignins of both coniferyl and sinapyl alcohol, and grass lignins of all three types [6]. The high complexity and inhomogeneity of the lignin structure is, in many cases, even further increased by currently applied pretreatment technologies and adds additional challenges for lignin’s downstream processing and valorization [7,8]. Compared to other pretreatment technologies, the organosolv process, used in this work, extracts relatively pure, low-molecular-weight lignin from biomass. This lignin shows a minimum of carbohydrate and mineral impurities and facilitates lignin applications with higher value than heat and power generation [9].

An approach to overcome these issues of high complexity and inhomogeneity is the production and application of nanostructured lignin. Nanostructured materials, especially in the 1–100 nm range, offer unique properties due to their increased surface area [10], while their important chemical and physical interactions are governed by surface properties. Hence, a nanostructured material can have considerably different properties than a larger-dimensional material of the same composition [11]. Therefore, the preparation of lignin nanoparticles and other nanostructures has gained interest among researchers during the last years.

The application fields of lignin nano- and microparticles are ranging from improvement of mechanical properties of polymer nanocomposites [10,11,12,13,14], bactericidal [11] and antioxidant properties [15,16] and impregnations [17] to drug carriers for hydrophobic and hydrophilic substances [18,19,20,21,22,23,24]. Also, a carbonization of lignin nanostructures can lead to high-value applications such as use in supercapacitors for energy storage [25]. Also, first attempts for the upscaling of the production process are under investigation [26]. However, most production methods published so far have a very high solvent consumption in common. Vast amounts of solvents are needed for purification of the lignin prior to precipitation, the precipitation itself and the downstream processing [27].

The degree of supersaturation is crucial in a precipitation process and has significant influence on the precipitation mechanisms and particularly on nucleation [28]. Therefore, the particle size varies with varying supersaturation. Furthermore, nucleation and growth kinetics of soluble substances are often greatly influenced by the hydrodynamic conditions prevailing during the crystallization process. The faster the solutions are thoroughly mixed, the higher the precipitation rate, the smaller the average particles size and the narrower the particle size distribution [29]. The speed of mixing is influencing the supersaturation of the lignin and therefore the nucleation rate. The nucleation rate is exponentially dependent on the supersaturation and must be considered as local phenomena. These local phenomena, however, can only be investigated in depth by computational fluid dynamic simulations [28].

So far, only one concept for production of micro- and nanoscale lignin was shown in an integrated lignocellulosic biorefinery in literature applying steam pretreatment, enzymatic hydrolysis, extraction of lignin with dimethyl sulfoxide and subsequent precipitation of nanoparticles in a dialysis process [30]. The present work also represents a concept for an integrated biorefinery and aims a direct precipitation of lignin in micro- to nanoscale from wheat straw organosolv pretreatment extracts. The organosolv pretreatment parameters were fixed while comparing different precipitation setups and conditions in order to identify the most suitable precipitation process for production of micro- to nanoscale lignin particles. The precipitation method used combines the most commonly used methods solvent shifting and pH shifting and reduces the solubility of the lignin by decreasing the solvent concentration and lowering of the pH value [27]. The three investigated precipitation setups allow different degrees of local supersaturation and therefore varying particle sizes. The size of the gained particles was measured both in the precipitation liquid and in water in order to investigate the influence of impurities on the particle size. The lignin particles were further investigated regarding their surface characteristics in terms of their ζ-potential, the precipitation yields, molecular mass and chemical structure. Furthermore, the most promising setup was tested at improved precipitation conditions to gain even smaller particles.

## 2. Results and Discussion

### 2.1. Composition of Wheat Straw Organosol Extract

The organosolv pretreatment utilizes mixtures of water and organic solvents at elevated temperatures in order to rupture the lignin-carbohydrate complex [31,32]. In this case, a 60 *w*/*w* % aqueous ethanol solution was applied at a temperature of 180 °C and resulted in an extract with the composition shown in Table 1. The applied parameters resulted in a lignin concentration of 6.62 g/L which can be distinguished between acid insoluble lignin and acid soluble lignin at a ratio of 5.1:1. The organosolv process is known to solubilize hemicellulose and lignin but the cellulose remains as solid [32]. In terms of concentration in the extract, the process shows a high selectivity for lignin compared to carbohydrates evident in the lignin to carbohydrate ratio of 9.8:1.

### 2.2. Particle Sizes and Behavior

Three different setups were used for precipitation: (1) “Batch” setup consisting of a stirred vessel filled with extract and addition of antisolvent; (2) “T-fitting” setup consisting of a T-fitting in which extract and antisolvent are mixed; (3) “static mixer” setup consisting of a T-fitting followed by static mixing elements in which extract and antisolvent are mixed. The speed of mixing in the different setups can be put in following order where the Batch setup presents the lowest speed of mixing, the T-fitting the intermediate speed of mixing and the static mixer the highest speed of mixing.

The lignin containing organosolv extract is a complex mixture of compounds in which only the major compounds are identified and quantified. In this process, compounds besides the lignin must be considered as impurities. But even trace amounts of impurities can have a significant effect on the precipitation process as a whole [33] including the aggregation behavior of the precipitated particles due to the influence of the impurities on the electrical double layer [34]. To investigate the influence of the sum of impurities on the particle size and width of the distribution, particles separated by centrifugation were on the one hand measured dispersed and diluted in supernatant from the centrifugation and on the other hand measured after the drying process dispersed in water using laser diffraction in both cases. The particles measured in supernatant are considered as intermediate and dried particles dispersed in water as end-product in the following discussion.

**Figure 1:**
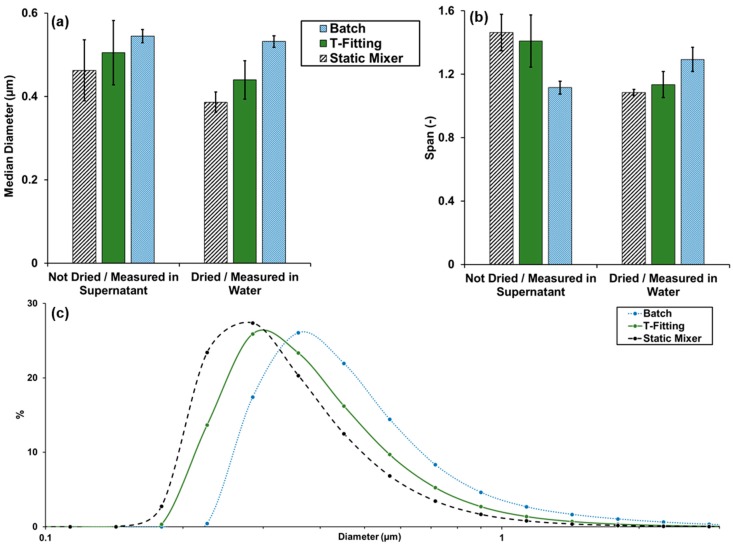
(**a**) Median diameters and (**b**) span of the undried particles measured in supernatant (intermediates) and dried particles dispersed in water (end-products). (**c**) Average particle size distributions of lignin particles precipitated with antisolvent with a pH of 2, dried and dispersed in water. All data from laser diffraction measurements. Points in the distributions curves indicate the center of the particles size class.

Figure 1 shows the average particle size distributions, median diameters and the span of the particle size distributions of the intermediate and end-product using different precipitation setups. Intermediate and end-product show a decreasing median particle diameter with increasing speed of mixing. Therefore, particles precipitated in the static mixer resulted in the smallest particle size. Furthermore, the centrifugal separation and drying of the particles after precipitation do not seem to influence the particle size negatively. For example, the particle size of the static mixer decreased from 463 ± 73 nm to 386 ± 24 nm after centrifugation and drying. The mode of the distribution curves for the setups T-fitting and static mixer is present in the particle size class from 252 nm to 317 nm. This class contains 25.9 ± 3.6% and 27.4 ± 1.2% of the number of particles for the T-fitting and Static mixer setup, respectively. The batch setup shows the mode in the particle size class from 317 nm to 399 nm containing 26.1 ± 0.6% of the particle number. The achieved particle sizes are in good agreement with other solvent shifting methods like the dialysis method given by Lievonen et al. [35] with particle sizes between 200 to 500 nm. Qian et al. [36] achieved particle sizes of 180 to 200 nm with a similar method compared to the batch method. However, these methods achieve the results by applying pure solvents and lignin which was already separated from the pretreatment or extraction solvents which might indicate a lower concentration of impurities compared to the method shown in this work.

On the other hand, the width of the distribution indicated by the span value shows contradictive results compared to the median diameter. The intermediate gained with batch precipitation shows the lowest span and, therefore, the narrowest distribution in this comparison whereas the end-product of the batch precipitation shows an increased span. The other two precipitation setups on the contrary show a narrower distribution after centrifugation and drying. A reason might be agglomeration and aggregation which are determined by the combination of several factors, which are dependent on the particle size and surrounding fluid. For particles smaller than 0.5 µm, the Brownian motion is dominant and leads to a so-called perikinetic collision mechanism which is influencing the agglomeration [34]. The yielded lignin particles cross the 0.5 µm threshold and, thus, different particle behavior might be expected. The exchange of the surrounding fluid by separation, drying and dispersing the particles in water is influencing the electrical double layer of the particles and therefore, the attractive and repulsive forces between the particles [34].

**Figure 2:**
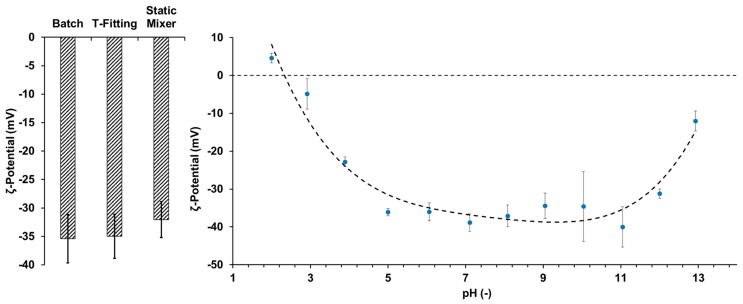
ζ-Potentials of lignin particles dispersed in pure water (**left**) and the pH-dependency of the ζ-potential of particles yielded in the static mixer (**right**).

To investigate this behavior, ζ-potential measurements were conducted for particles from all precipitation setups. Figure 2 (left) shows the ζ-potentials of the lignin particles in ultra-pure water—all of which are in the magnitude of −30 to −40 mV indicating a low tendency of agglomeration under these conditions. A *t*-test showed significant (confidence coefficient 95%) difference between the ζ-potential of particles from the static mixer compared to the Batch and T-fitting setup, respectively. Particles from the Batch and T-fitting showed no significant difference.

Particles from the static mixer were further investigated at pH values from 2 to 13 (see Figure 2 right). The lowest ζ-potentials and therefore, the highest stability of the particles was observed in the pH range from 5 to 11. Below a pH value of 5 the ζ-potential is increasing, indicating instable particles and a higher tendency of agglomeration. At elevated pH values above 11, the ζ-potential is also increasing and dissolution of the lignin was observed.

**Figure 3:**
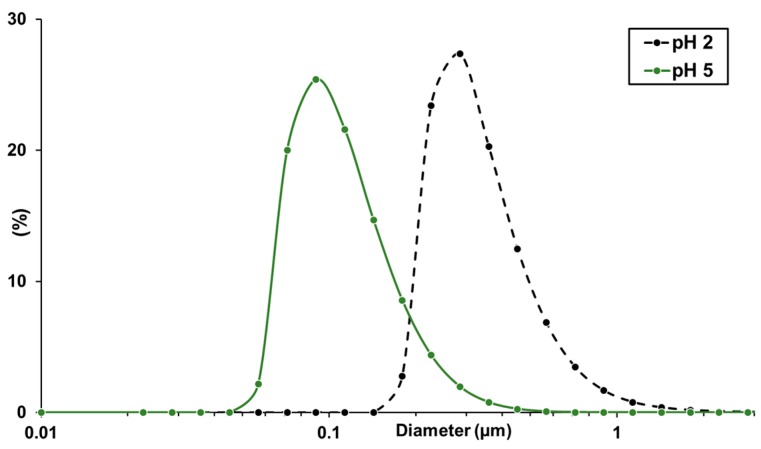
Average particle size distributions of lignin particles dispersed in water measured via laser diffraction from precipitations in the static mixer applying antisolvents with pH values of 2 and 5.

Based on the results from the pH-dependent ζ-potential measurements, additional precipitation experiments were carried out for the precipitation set up with the static mixer, as it yielded the smallest particles, with a pH-5 antisolvent. Figure 3 compares the particle size distributions of particles precipitated in the static mixer applying antisolvent with a pH value of 2 and 5, respectively. The organosolv extract used exhibits a pH of 5.7. While the precipitation with pH 2 antisolvent drops the pH value of the resulting suspension to around 2.3, the use of pH 5 antisolvent retains the pH value almost constant and, therefore, in a pH region with a lower ζ-potential and less tendency for agglomeration. This is resulting in a median diameter significantly reduced by a factor of 3.

**Figure 4:**
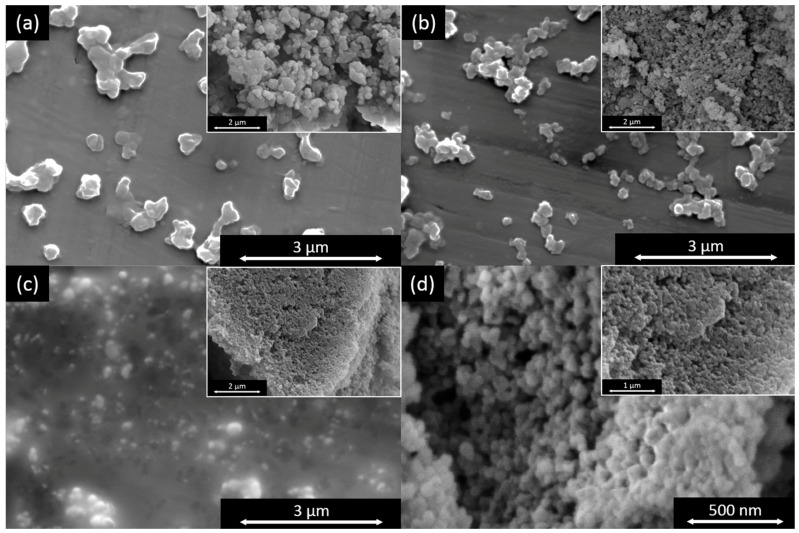
Scanning electron microscopy (SEM) images of dried lignin particles from different precipitation set-ups. Full-size images show particles after dispersion in Hexane and the small-scale pictures particles directly after centrifugation and drying; (**a**) batch with antisolvent pH 2; (**b**) T-fitting with antisolvent pH 2; static mixer with antisolvent pH 2 (**c**) and pH 5 (**d**).

The scanning electron microscopy images of the dried lignin particles shown in Figure 4 confirm the tendency shown by the laser diffraction results. The largest particles can be seen in the image of the batch precipitation setup and the smallest from the static mixer using antisolvent with pH 5.

### 2.3. Yield

To ensure an efficient process, precipitation yields were investigated. The yields shown are defined as the ratio of the lignin amount in the utilized volume of organosolv extract to the mass of precipitate after drying in the climatic chamber.

**Figure 5:**
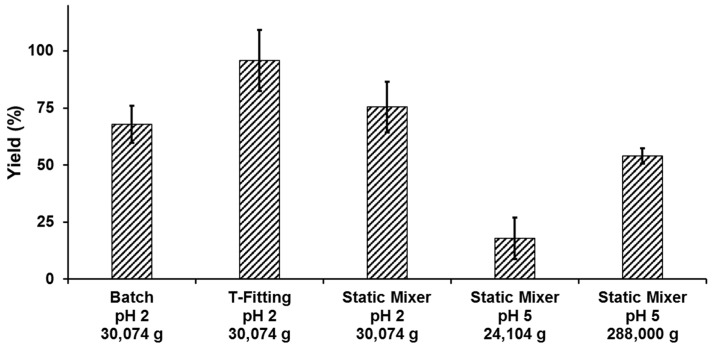
Precipitation yields of the different precipitation setups, antisolvent pH values and *g*-forces.

For precipitation with pH 2 antisolvent, the average mass loss during the drying process was 90.8 ± 2.1 wt %, 89.9 ± 2.5 wt % and 91.6 ± 1.5 wt % for the batch, T-fitting and static mixer setup, respectively. The precipitation yields are shown in Figure 5. A t-test shows a significant (confidence coefficient 95%) higher yield of the T-fitting compared to the static mixer and Batch setup, respectively. While a thoroughly mixing of extract and antisolvent is ensured in the batch and static mixer setup, this cannot be guaranteed in the T-fitting. This might result in a final particle formation in the non-stirred collection vessel and causing a difference in yield.

Precipitations conducted in the static mixer with antisolvent at a pH value of 5 show significantly lower yields compared to pH 2 precipitations when separated at 24,104× *g*. By increasing the g-force to 288,000× *g* in an ultracentrifuge, the yield was increased to over 50 wt % but it was still lower compared to pH 2 precipitations. However, increasing g-forces to over 30,074× *g* did not increase the yield for particles from precipitations with a pH-value of 2.

The yields are in a reasonable range comparing to the results of other methods. For example Tian et al. [30] could reach yields between 41.0% and 90.9% using a dialysis method applying dimethyl sulfoxide as solvent for poplar, lodgepole pine and corn stover lignin and water as antisolvent. Furthermore, this work represents the most comparable process found in literature since a whole process chain from the raw material to the final lignin particles including impurities was shown. Yearla et al. [37] showed a process with yields from 33% to 63% by adding lignin/acetone/water mixtures rapidly in water. On the other hand the presented methods show much higher yields than the method used by Yang et al. [10], which has a yield only around 10% applying ethylene glycol as solvent for lignin and hydrochloric acid to reduce the pH-value and therefore the solubility of the lignin.

### 2.4. Lignin Properties

The investigation of the properties of the lignin particles is rather dedicated to the lignin resulting from the whole biorefinery concept than to differences due to different precipitation setups and operating parameters since the results show only minor influence on the variations.

**Figure 6:**
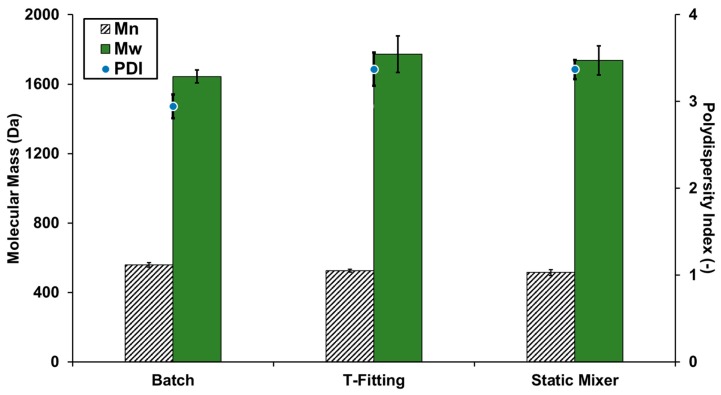
Molecular mass of precipitated lignin in the different set-ups with pH 2 antisolvent.

The lignin properties were investigated using high performance size-exclusion chromatography (HPSEC) for the determination of the molecular mass and attenuated total reflection Fourier transform infrared spectroscopy (ATR-FTIR) to acquire basic information about the chemical structure of the particles. The results of the molecular mass determination are shown in Figure 6. The results show only little influence of the precipitation setup on the molecular mass of the lignin.

**Figure 7:**
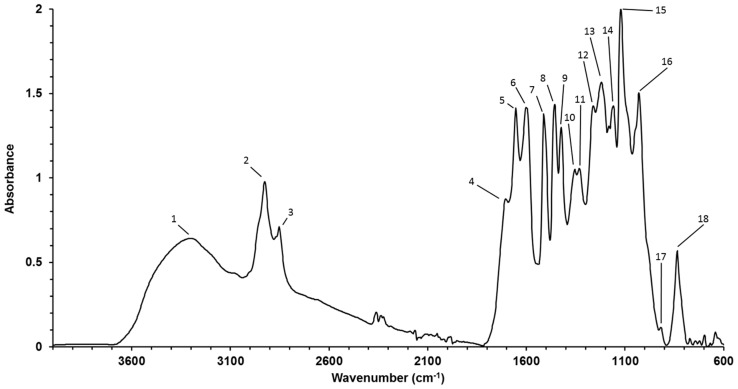
Average FTIR-spectra of lignin precipitated in the static mixer with antisolvent at a pH-value of 2.

The average spectra of particles resulting from the static mixer setups are shown in Figure 7. The average lignin spectra from the three different setups show only minor differences and it can be assumed that the speed of mixing has only minor influence on the chemical composition of the resulting particles. Bands of the spectra are assigned in Table 2 and adapted from Faix et al. [38]. Wheat straw lignin consists of all three major types of monolignols: coniferyl alcohol (G-units), coumaryl alcohol (H-units) and sinapyl alcohol (S-units), and is therefore classified as HGS-type lignin [6]. The samples have bands at 1331 cm^−1^, 1262 cm^−1^, 836 cm^−1^ (C H out-of-plane in positions 2 and 6 of S units, and in all position of H units), and 1160 cm^−1^ (typical for HGS) which matches the results from other investigations of HGS type lignins [39]. Aromatic skeleton vibrations occur at 1603, 1513 and 1424 cm^−1^, in which the aromatic semicircle vibration (a vibration involving both C–C stretching and a change of the H–C–C bond angle) is assigned at 1513 cm^−1^ [40].

## 3. Materials and Methods

### 3.1. Materials

The wheat straw used was harvested in 2015 in lower Austria and stored under dry conditions until use. The particle size was reduced in a cutting mill, equipped with a 5-mm mesh, before pretreatment. The composition of the straw was 16.1 wt % lignin and 63.1 wt % carbohydrates consisting of Arabinose, Glucose, Mannose, Xylose and Galactose. Ultra-pure water (18 MΩ/cm) and Ethanol (Merck, Darmstadt, Germany, 96 vol %, undenaturated) was used in the organosolv treatment and additionally sulfuric acid (Merck, 98%) in the precipitation steps.

### 3.2. Experimental Procedure

**Figure 8:**
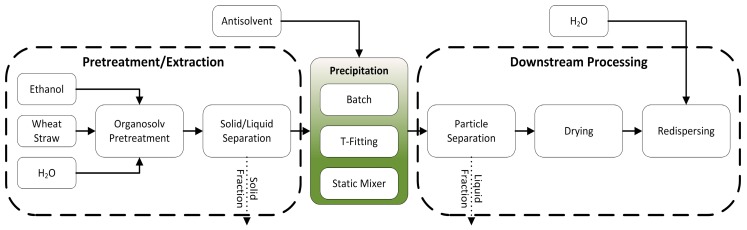
Schematic representation of the experimental procedure indicating the three main parts “Pretreatement/Extraction”, “Precipitation” and “Downstream Processing”.

The experimental work is structured in three parts, illustrated in Figure 8. In the “Pretreatment/Extraction” part, the lignin is extracted from the wheat straw applying an organosolv process and solids are removed from the extract. Three different precipitation setups are applied in the “Precipitation” part in order to achieve varying mixing behaviors of lignin containing extract and antisolvent. In the “Downstream Processing”, resulting particles are separated from the suspension via centrifugation, dried and dispersed in water. Lignin particles dispersed in water are denoted as “end product”.

#### 3.2.1. Pretreatment/Extraction

The organosolv pretreatment was conducted in a 1 L stirred autoclave (Zirbus, HAD 9/16, Bad Grund, Germany) using a 60 wt % aqueous ethanol mixture as solvent under consideration of the water content in the straw. The wheat straw content in the reactor based on dry straw was 8.3 wt %. The reactor was heated to 180 °C within 45 min and held at this temperature for 15 min. After these 60 min of treatment, the reactor was cooled to room temperature. The solid and liquid fractions were separated using a hydraulic press (Hapa, HPH 2.5, Achern, Germany) at 200 bar and a centrifuge (Thermo Scientific, Sorvall, RC 6+, Waltham, MA, USA) at 30,074× *g* for 20 min. The supernatants of the single batches were unified, the composition was analyzed, and it was stored at 5 °C until the precipitation experiments were performed.

#### 3.2.2. Precipitation

**Figure 9:**
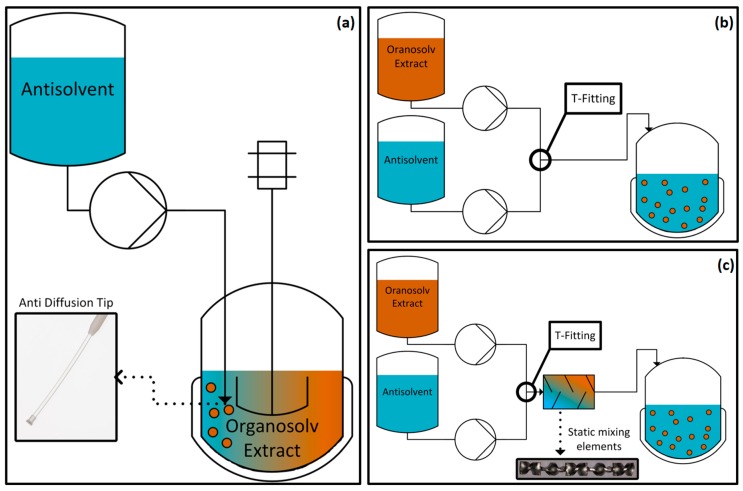
Schematic illustration of the three different precipitation setups; (**a**) Batch precipitation where antisolvent is added to the extract; (**b**) mixing of extract and antisolvent in a T-fitting; (**c**) mixing of extract and antisolvent in a T-fitting followed by a static mixer.

The precipitation experiments of the lignin dissolved in the organosolv extract were conducted at 25 °C. The volume ratio of extract to antisolvent was kept constant at 1:5, where the antisolvent consisted of water set to the desired pH value with sulfuric acid. Three different setups (see Figure 9) that vary in the degree of mixing and therefore, the degree of supersaturation, were compared. All results are shown as average values of the repetitions for each precipitation setup including standard deviation.

Setup (a), denoted as “Batch”, consists of a temperature-controlled 250-mL beaker with a magnetic stirrer and a syringe pump for addition of the antisolvent via an antidiffusion tip. 20 mL extract were placed in the beaker and 100 mL antisolvent were added with a flow rate of 20 mL/min through the immersed anti-diffusion tip. The stirrer speed was kept constant at 625 rpm. The Batch precipitation was repeated 5 times.

Setup (b), denoted as “T-fitting”, consists of two syringe pumps for extract and antisolvent connected to a T-fitting and a 20.4 cm long pipe with inner diameter of 3.7 mm. At the end of the pipe, a metering valve was placed and connected to a beaker with a 1-m rubber hose with an inner diameter of 4 mm. The whole setup was temperature controlled and the precipitate was collected in a temperature controlled 250 mL beaker without stirring. Extract and antisolvent were added with a flow rate 4 mL/min (20 mL total volume) and 20 mL/min (100 mL total volume), respectively.

Setup (c), denoted as “Static Mixer”, is identical to setup (b) but the pipe after the T-fitting is equipped with 6 static mixing elements (Striko, Helical DN4) each 32 mm in length. The 6 combined elements redirected the flow 30 times which results in a mixing degree of 99% based on information delivered by Striko. The static mixing elements were placed directly after the T-fitting with 26.7 mm between the center of the T-fitting and the first static mixing element. Total volume and flow rates were identical to setup (b).

#### 3.2.3. Downstream Processing

The resulting lignin suspension was centrifuged immediately after precipitation with a Thermo Scientific, Sorvall RC 6+ centrifuge at 30,074× *g*, a Sigma 4k15 centrifuge at 24,104× *g* or a Thermo WX Ultra 80 ultracentrifuge (Thermo Scientific, Waltham, MA, USA) at 288,000× *g* for 20 min to separate the lignin particles. The supernatant was decanted and collected. The precipitate was divided, where a small amount was dispersed in supernatant in order to achieve a suitable dilution for measurement of the particle size distribution via laser diffraction. The major amount was dried in a climatic chamber at a temperature of 40 °C and a dew point of 0 °C for at least 24 h. Dry particles were investigated by using scanning electron microscopy (SEM). Finally, the dry particles were dispersed in water applying sonication and particle size distribution was measured.

### 3.3. Analytics

The organosolv extract was analyzed for carbohydrates, lignin and degradation products. The carbohydrate content was determined by the sample preparation following the National Renewable Energy Laboratory (NREL) laboratory analytical procedure (LAP), “Determination of Sugars, Byproducts, and Degradation Products in Liquid Fraction Process Samples” [41], but no neutralization of the samples after hydrolysis was conducted and a Thermo Scientific ICS-5000 HPAEC-PAD system (Thermo Scientific, Waltham, MA, USA) with deionized water as the eluent was used for the determination of arabinose, glucose, mannose, xylose and galactose. The concentration of the degradation products acetic acid, HMF and furfural were determined with a Shimadzu LC-20A “prominence” HPLC system and a Shodex SH1011 column (Showa Denko, Tokyo, Japan) at 40 °C, with 0.005 M H_2_SO_4_ as eluent. The lignin content was determined following the NREL LAP “Determination of Structural Carbohydrates and Lignin in Biomass” [42] using the dry matter of the extract obtained at 105 °C.

Dried precipitate was investigated in terms of molecular mass, chemical composition via ATR-FTIR and via scanning electron microscopy (SEM). The molecular weight was measured via high performance size-exclusion chromatography (HPSEC) using an Agilent 1200 HPLC system equipped with a UV detector. The lignin samples were dissolved in 10 mM NaOH and compared with sodium-polystyrene-sulfonate standards ranging from 210 Da to 77 kDa. The ATR-FTIR absorption data were obtained by using a Vertex 70 Fourier Spectrometer (Bruker, Billerica, MA, USA) in spectral range 600–4000 cm^−1^ (resolution 4 cm^−1^). The lyophilized lignin powder was pressed against the diamond crystal of the ATR device. A pressure applicator with a torque knob ensured that the same pressure was applied for all measurements. For each lignin sample, 32 scans were acquired and averaged. Background scanning and correction was carried out after each sample. For each sample, four different subsamples were measured, and the resultant mean spectra were used for further analyses. The baseline was corrected using the rubber band method and the spectra normalized on the minima and maxima of the spectra. The particle size and shape were investigated in a SEM (Fei, Quanta 200 FEGSEM). The samples were sputter coated with 4 nm Au/Pd (60 wt %/40 wt %) before analysis.

The ζ-potential was investigated with a ZetaPALS (Brookhaven Instruments, Holtsville, NY, USA). Dried particles were dispersed in water at an approximate concentration of 20 mg/L and aged 24 h before the measurement. Each measurement was composed of 5 runs with each 30 sub runs and was conducted at 25 °C.

Particle size measurements were conducted in a Mastersizer 2000E (Malvern Instruments, Malvern, UK) using laser diffraction in liquid suspensions. The refractive index of the particles was set to 1.53 and the absorption to 0.1. Two different measurements were conducted: (1) Centrifuged and not dried particles dispersed in supernatant, (2) Centrifuged and dried particles dispersed in water. The refractive index of the supernatant was measured for each experiment as input for the Mastersizer 2000E software. A standard operation procedure was developed including sonication and stirrer speed of 2000 rpm in the dispersion unit and 15-min equilibration time before the measurement. Each measurement consisted of 5 single measurements in 5 s intervals. The span is the measurement of the width of the particle size distribution defined as:

(1) $$Span=\frac{d_{0.9}-d_{0.1}}{d_{0.5}}$$

$d_{0.9}$ and $d_{0.1}$ are the particle sizes below which 90% and 10%, respectively, of the sample lies. $d_{0.5}$ is the median diameter. The narrower the distribution, the smaller the span becomes. All reported distributions are particle number based distributions.

## 4. Conclusions

Three different setups for the precipitation of micro- to nanoscale lignin particles from organosolv wheat straw extracts were compared in a biorefinery process chain from raw material to the final product. Precipitation by mixing lignin-containing extract with antisolvent in a T-fitting followed by a static mixer represents the setup with the highest speed of mixing in the present comparison and results in the smallest particles. The median particle sizes decrease from 532 ± 14 nm to 386 ± 24 nm compared to a batch precipitation setup at comparable precipitation parameters. Shifting the pH value of the antisolvent from 2 to 5 decreased the resulting median particle diameter significantly to almost 100 nm. Drying of particles did not influence the particle sizes negatively by showing decreased particle diameters after the separation process. The precipitation setups, Batch and Static Mixer, showed comparable precipitation yields applying pH 2 antisolvent whereas the T-fitting showed significantly higher precipitation yields. Furthermore, the yield was significantly influenced by the pH of the antisolvent where the lower pH values of 2 increased the yield compared to pH 5. The molecular mass of the lignin particles and their chemical composition was not influence by the precipitation setups. It can thus be concluded that the precipitation of lignin nanoparticles should preferably be carried out with a T-fitting and static mixer. If particle sizes should be minimized, the antisolvent with pH 5 is superior to pH 2. In contrast, if yields should be optimized, the antisolvent pH 2 is superior to pH 5.

## Figures and Tables

**Table 1:** Composition of an organosolv extract at the used pretreatment conditions.

Compound/Property	Value	Unit
Ethanol	511	g/L
Total Carbohydrates ^1^	0.677	g/L
Monomeric Carbohydrates ^1^	0.201	g/L
Acetic Acid	1.43	g/L
Acid Insoluble Lignin	5.53	g/L
Acid Soluble Lignin	1.09	g/L
pH	5.7	-
Density ^2^	0.901	g/mL
Dry Matter ^3^	1.57	*w*/*w* %

^1^ Sum of arabinose, galactose, glucose, xylose and mannose concentrations; ^2^ at 25 °C; ^3^ determined at 105 °C.

**Table 2:** Assignments of FTIR absorption bands. Adapted from Faix.

Number in Figure 8	Band (cm^−1^)	Assignment
1	3302	O–H Stretch
2	2926	C–H Stretch in methyl and methylene groups
3	2852	C–H Stretch in methyl and methylene groups
4	1706	C=O stretching
5	1654	C=O Stretch; in conjugated *p*-subst. aryl ketones
6	1603	aromatic skeletal vibrations plus C=O Stretch
7	1513	Aromatic skeletal vibrations
8	1457	Asymmetric bending deformation of methyl and methylene groups
9	1424	aromatic skeletal vibrations combined with C–H in-plane deform
10	1355	aliphatic C-H stretch in CH_3_
11	1331	S ring and G ring condensed
12	1262	C–O of guaiacyl ring and C=O stretch
13	1220	C–C plus C–O stretch
14	1160	Typical for HGS lignin, conjugated C=O in ester groups
15	1122	aromatic C–H in-plane deformation (typical for S units), plus secondary alcohols, plus C=O stretch
16	1031	Aromatic C–H in-plane deformation, G > S; plus C–O deform, in primary alcohols; plus C=O Stretch (unconjugated)
17	919	C–H out-of-plane; aromatic
18	836	C–H out-of-plane in position 2 and 6 of S, and in all position of H units
